# Fiber alignment drives changes in architectural and mechanical features in collagen matrices

**DOI:** 10.1371/journal.pone.0216537

**Published:** 2019-05-15

**Authors:** Paul V. Taufalele, Jacob A. VanderBurgh, Adam Muñoz, Matthew R. Zanotelli, Cynthia A. Reinhart-King

**Affiliations:** 1 Department of Biomedical Engineering, Vanderbilt University, Nashville, Tennessee, United States of America; 2 Nancy E. and Peter C. Meinig School of Biomedical Engineering, Cornell University, Ithaca, New York, United States of America; University of California Berkeley, UNITED STATES

## Abstract

Aligned collagen architecture is a characteristic feature of the tumor extracellular matrix (ECM) and has been shown to facilitate cancer metastasis using 3D in vitro models. Additional features of the ECM, such as pore size and stiffness, have also been shown to influence cellular behavior and are implicated in cancer progression. While there are several methods to produce aligned matrices to study the effect on cell behavior in vitro, it is unclear how the alignment itself may alter these other important features of the matrix. In this study, we have generated aligned collagen matrices and characterized their pore sizes and mechanical properties at the micro- and macro-scale. Our results indicate that collagen alignment can alter pore-size of matrices depending on the polymerization temperature of the collagen. Furthermore, alignment does not affect the macro-scale stiffness but alters the micro-scale stiffness in a temperature independent manner. Overall, these results describe the manifestation of confounding variables that arise due to alignment and the importance of fully characterizing biomaterials at both micro- and macro-scales.

## Introduction

The extracellular matrix (ECM) contains chemical and physical cues that guide cellular behavior [1]. During tumor progression, the tumor ECM becomes deregulated resulting in altered chemical and physical cues [2]. These ECM transformations contribute to abnormal cell behavior and ultimately help to drive cancer progression [2]. Thus, the ECM plays a critical role in cancer and it is important to fully understand its properties. Recently, attention has been drawn to the altered physical properties of the tumor ECM, as it has been an understudied aspect of cancer that has proven to display increasingly more control over cellular function [3]. Due to increased collagen deposition and cross-linking, tumors are characteristically stiffer than healthy ECM [4,5]. This enhanced matrix stiffness has been shown to regulate cellular proliferation [6], migration [7], and tissue morphogenesis [8] which have many implications in tumor growth [5] and metastasis [9]. In addition to increased matrix stiffness, excess collagen deposition leads to reduced pore sizes in the ECM [10,11]. Reduced pore sizes have been shown to hinder 3D cell migration [11] and may require cells to remodel the ECM via matrix degrading enzymes such as matrix metalloproteinases (MMPs) to navigate the ECM [12].

In addition to depositing and cross-linking matrix, cancer cells are also capable of remodeling collagen in the ECM to generate regions of highly aligned collagen fibers [13,14]. This feature is often seen at the tumor periphery[13] and has been identified as a prognostic marker in human breast cancer [15]. Aligned collagen matrices provide guidance cues for migrating cancer cells and promote migration direction persistence [14]. Furthermore, collagen alignment has been shown to reduce the energy required for cancer cell migration [16] and may facilitate intravasation *in vivo* during tumor progression [17]. While it is known that enhanced collagen deposition leads to a significantly stiffer ECM with smaller pore sizes, and collagen matrices can be stiffened via cross-linking without altering the network architecture, it is unclear how aligning collagen matrices affects other architectural and mechanical features. Stylianopoulos et al. computationally predict that pore sizes are larger in aligned regions while Ray et al. reports smaller pores in matrices aligned by cells [18,19]. Because architectural features and mechanical properties of the ECM are crucial regulating factors during tumor progression, it is important to understand their relationship relative to alignment. Moreover, previous work has shown that macro-scale properties, such as bulk density of collagen gels, may not accurately reflect the effective property that the cells experience at the micro-scale [10]. However, many studies report mechanical properties at either the micro- or macro-scale but not both [7,20–22]. Thus, we measured and compared the micro- and macro-scale mechanical properties of the collagen matrices.

In this study, we investigated the architectural and micro- and macro-scale mechanical properties between aligned and randomly oriented collagen matrices. We quantified matrix pore size as well as micro- and macro-scale mechanical properties of aligned collagen matrices compared to randomly oriented matrices. We used two different polymerization temperatures to account for confounding matrix parameters such as network architecture [23] and fibril morphology [11,24]. Our data indicate that collagen alignment significantly alters pore size in gels polymerized at higher temperatures. Mechanical characterization reveals that macro-scale stiffness is not affected by alignment or polymerization temperature while the micro-scale stiffness decreases as polymerization temperature increases. Together these findings reveal that collagen alignment can induce confounding architectural and mechanical differences that are also known to affect cell behavior, and macro-scale measurements of stiffness may not be reflective of stiffness at the micro-scale.

## Materials and methods

### Collagen gel preparation

Type I collagen was acid solubilized in 0.1% glacial acetic acid (Macron, V193-14) from rat tail tendons to obtain 10 mg/ml type I collagen stock solution. Each collagen gel was mixed as a separate solution of stock collagen diluted to 1.5 mg/ml with 0.1% glacial acetic acid, 10X HEPES buffer, 1X PBS, and neutralized with 1N NaOH. Gels were allowed to polymerize at 37°C for 1 hr or 25°C for 1.5 hr prior to usage.

Collagen gels were loaded into a custom polydimethylsiloxane (PDMS) devices, as previously described [25]. To create the custom PDMS device used for collagen matrix alignment, a 15 mm x 15 mm x 5 mm PDMS square was formed, from which a 10 mm x 10 mm section was then removed (S1 Fig). A no. 1.5 glass slide was attached to the front side of the PDMS mold using silicon to enclose the 10 mm opening and create the fourth wall of the chamber (S1 Fig). The PDMS molds were then attached to large glass slides using vacuum grease to seal the bottom of the chambers onto the glass slide. To achieve collagen alignment, paramagnetic polystyrene beads (PM-20-10; Spherotech, Lake Forest, IL) were incorporated into a collagen solution at 1% (vol/vol). Collagen solution containing paramagnetic polystyrene beads was loaded into the custom PMDS device and placed next to a neodymium magnet (BZX0Y0X0-N52; K&J Magnetics, Pipersville, PA) while the collagen polymerized. Collagen gels without paramagnetic polystyrene beads were created to serve as randomly-oriented controls.

### Confocal reflectance microscopy

Collagen fiber architecture was visualized via confocal reflectance using a Zeiss Axio Examiner.Z1 equipped with a LSM700 confocal module using a 405-nm laser, and a W Plan-Apochromat 20x/1.0 N.A. water immersion objective operated by Zen 2010 software. Images were taken throughout the gels and at least 150 μm above the glass-gel interface.

### Analysis of collagen microstructure

Collagen fiber orientation was analyzed in ImageJ using the Orientation J plugin to generate pseudocolor visual representations and fiber orientation distributions. An orientation index was generated from the orientation distribution by implementing a previously described method as a custom Matlab script [26]. In brief, the orientation index, S, is defined by

$$S=2<{cos}^{2}\alpha >-1$$ (1)

where α represents the angle between an individual fiber and the average fiber orientation and <*cos*^2^α> represents the averaged square cosine of all α per image. An orientation index of 0 represents a perfectly random distribution, and an orientation index of 1 represents a perfectly aligned distribution. To further quantify fiber alignment, a custom Matlab script was used to assess anisotropy based of the Fourier transform of confocal reflectance images. In brief, the 2D fast Fourier transform was computed for each image and an ellipse was fit to the subsequent power spectrum. A measure of anisotropy was obtained by calculating the aspect ratio of the fit ellipse from the long and short axes.

To measure pore size from confocal reflectance images, two methods were employed as custom Matlab scripts (MathWorks, R2018a). The 2D autocorrelation function in Matlab was used to quantify the characteristic pore size in an image as previously described [10]. Images were uploaded into Matlab and preprocessed to remove background noise using an adaptive Weiner filter (0.625 μm filtering window) and a TopHat filter (0.94 μm strel disk diameter) and finally converted to a binary image. The 2D autocorrelation was computed for each image and the characteristic pore size was derived from the decay measured in the autocorrelation. An erosion-based algorithm was also used to measure pore size, as described previously [27]. In brief, confocal images were uploaded into Matlab and preprocessed to remove background noise as described above. Images were then converted to binary and eroded with progressively larger disk sizes until a threshold of 50% image erosion was crossed. Clusters of adjacent pixels with the same value were grouped together and labeled as objects. The objects containing ‘on’ pixels represented pores, while the objects containing ‘off’ pixels represented collagen fibers. The area of each object representing a pore (objects containing ‘on’ pixels) was measured and the average area was used to calculate an average pore diameter.

To measure fiber diameter from confocal reflectance images, we adapted a previous method utilizing line scans [28]. In brief, line scans were computed over confocal reflectance images and fiber diameter was determined for each image as the average peak width at half prominence.

### Macro-Scale stiffness

Macro-scale stiffness was determined by confined compression as previously described [29]. Collagen gels were loaded onto a TA Electroforce Model 3100 (TA Instruments) that performed 5% stepwise indentations and used a 250g load cell to measure the resulting forces. The stress relaxation data was then fit to a standard linear solid model of viscoelastic behavior via a custom Matlab script. The equilibrium modulus was then calculated from the slope of the resulting stress-strain curve.

### Micro-Scale stiffness

The micro-scale stiffness was determined by atomic force microscopy (AFM). The Young’s modulus of each collagen gel was measured using AFM in contact mode (MFP-3D, Asylum Research, CA). Indentations were performed at a minimum of 3 regions within each collagen gel. Force-displacement curves were taken at 30 points within each region within a 120 by 120 μm grid (6 x 5), for a total of 90 indentations for each collagen gel. Indentations were made at a loading rate of 1 μm/s and trigger force of 2 nN with silicon nitride cantilevers with a nominal spring constant of 0.01 N/m and a 4.5 μm diameter spherical polystyrene bead (Novascan, Boone, IA). AFM tips were calibrated before use and had a mean spring constant of 0.015 ± 0.002 N/m. Force-displacement curves were fit to the Hertz model assuming a Poisson’s ratio of 0.5 using the Asylum curve fitting software to determine the elastic modulus.

### Statistical analysis

Statistical analyses were performed using GraphPad Prism 7.0 (GraphPad Software, La Jolla, CA, USA). Ordinary two-way ANOVA followed by Tukey’s multiple comparison test were performed on all image analysis and macro-scale mechanical testing results. The non-parametric Kruskal-Wallis test followed by Dunn’s multiple comparison test was applied to the micro-scale mechanical testing. ‘N’ represents the number of independent samples while ‘n’ represents the number of measurements taken.

## Results

### Temperature alters the degree of collagen alignment

**Fig 1:**
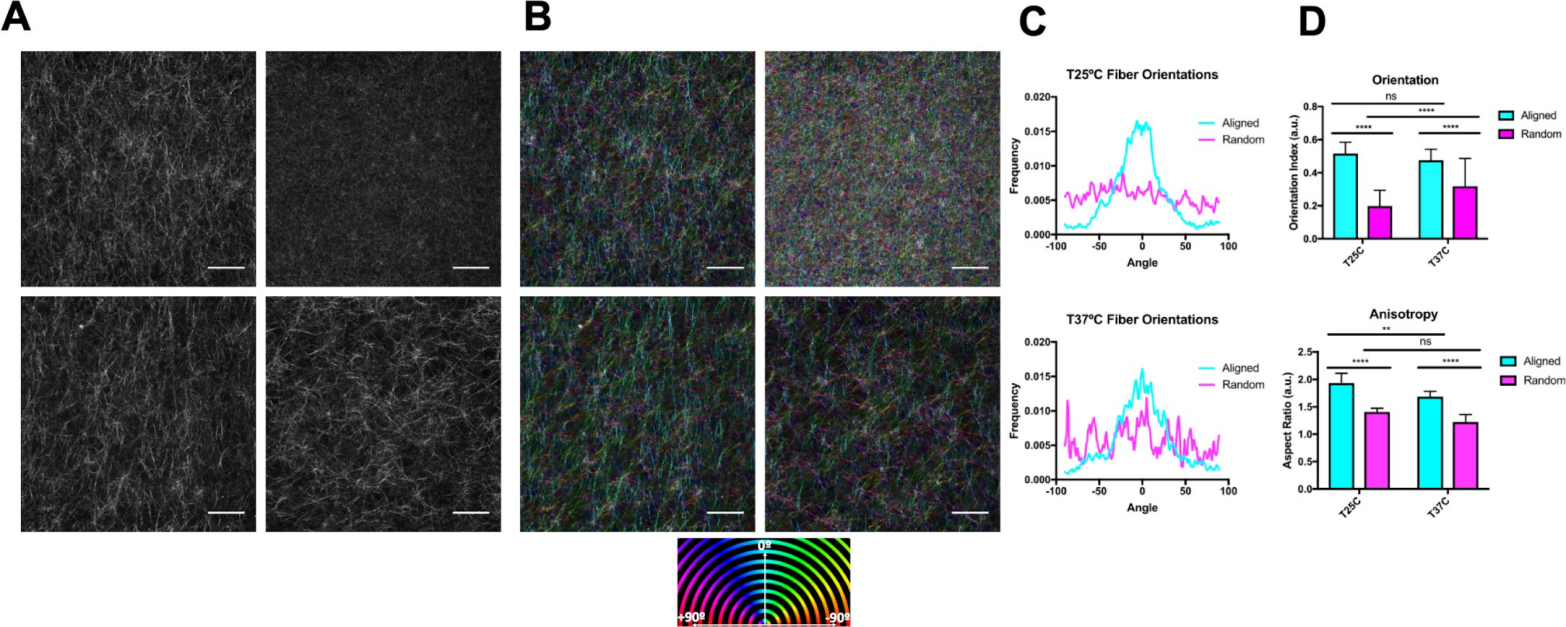
The effects of temperature on collagen alignment. (A) Representative confocal reflectance images. (B) Pseudo-color confocal reflectance images depicting fiber orientations. The 0° mark indicates the direction the beads were pulled to induce alignment. (C) Representative histograms depicting fiber orientation distributions generated from the OrientationJ plugin in ImageJ. (D) Quantifications of the collagen alignment via 2 methods: aspect ratio and orientation index. N = 6–7; n = 36–42. Data presented as mean ± s.d.

Network architecture, specifically network connectivity, pore size, and fiber diameter, are heavily influenced by polymerization temperature [24,30]. By increasing polymerization temperature, others have shown an increase in network connectivity and decreases in pore size [24,30]. To investigate matrix alignment under varied network architectures, we characterized collagen matrices polymerized at 25°C and 37°C. To measure the alignment of the collagen matrices, confocal reflectance images were analyzed via the OrientationJ plugin in ImageJ (Fig 1A and 1B). Pseudocolor images generated by OrientationJ reveal strong coherency of fiber colors in the aligned matrices compared to the random matrices at both temperatures (Fig 1C). Furthermore, fiber orientation histograms show a robust peak around 0 degrees in the aligned collagen matrices compared to the random matrices at both temperatures (Fig 1C). The fiber orientation distributions were used to calculate an orientation index as described in the methods. To further confirm the alignment and provide a quantitative measure of alignment in each matrix, we calculated the aspect ratio of 2D Fourier transform spectra derived from confocal reflectance images. At both temperatures, the orientation index and aspect ratio were significantly higher in aligned matrices compared to random matrices indicating significant alignment occurred at both temperatures (Fig 1D). Interestingly, the aspect ratio of aligned collagen matrices is significantly higher at 25°C compared to aligned matrices at 37°C, indicating a higher degree of anisotropy at the lower temperature. However, there is no significant difference between the orientation indexes of aligned collagen matrices at 25°C and 37°C, indicating similar percentages of aligned fibrils at both conditions. Together, these data indicate that fiber alignment is possible at both temperatures but may be more perceptible at 25°C compared to 37°C.

### Collagen alignment alters pore size in a temperature dependent manner

**Fig 2:**
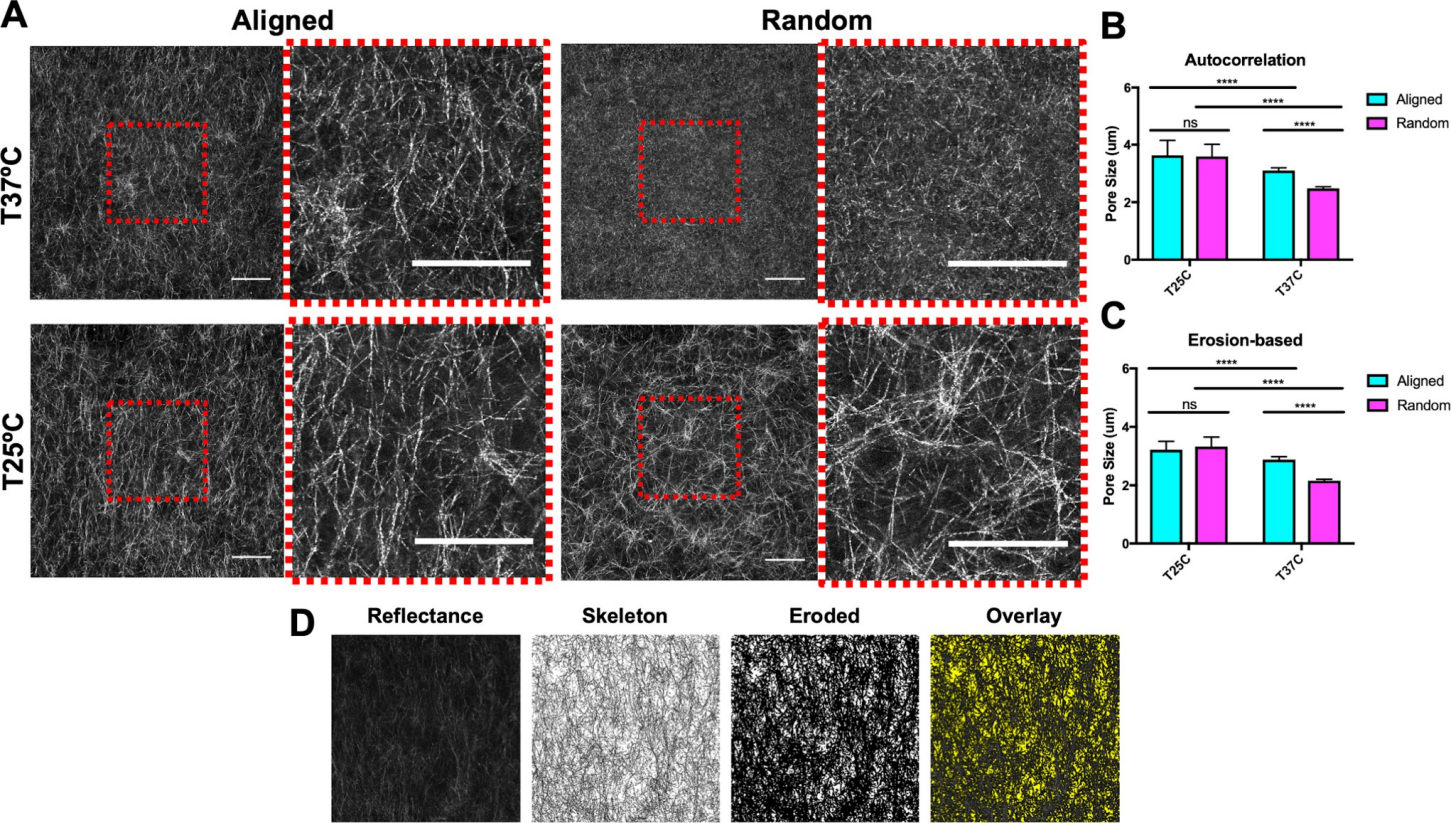
The effects of collagen alignment at different temperatures on pore size. (A) Representative confocal reflectance images of aligned & random collagen gels gelled at 25°C and 37°C. Cropped and magnified images are included to the right of the images. Scale bars = 50 μm. Collagen pore size quantified by autocorrelation methods (B) and erosion-based methods (C). N = 6–7; n = 36–42. Data presented as mean ± s.d. (D) Erosion-based quantification process. Representative confocal reflectance image of collagen architecture is transformed into a skeletonized binary image with black pixels depicting fibers. Pores are produced by erosion of the skeletonized binary image.

Prior studies have utilized temperature to control pore size of collagen matrices independently of collagen density [31] and have demonstrated that decreasing polymerization temperature induces larger pore sizes [11,31]. To investigate the effects of matrix alignment under different temperatures on collagen pore size, confocal reflectance images were captured (Fig 2A) and analyzed using custom Matlab scripts to quantify pore size. Here, we utilized both a 2D autocorrelation (Fig 2B) and erosion-based algorithm (Fig 2C and 2D) originally designed to quantify the microarchitecture of randomly aligned collagen matrices to ensure that our findings were robust as well as to mitigate any possible technical aberrations. As expected, the random gels polymerized at 25°C have significantly larger pores than random gels polymerized at 37°C (Fig 2B and 2C). Interestingly, there was no difference in pore size between aligned and random matrices at 25°C, whereas the aligned matrices had significantly larger pore size than the random matrices at 37°C (Fig 2B and 2C). These findings were evident in pore size measurements from both the autocorrelation and erosion-based methods.

### Collagen alignment decreases stiffness at the micro-scale but not at the macro-scale

**Fig 3:**
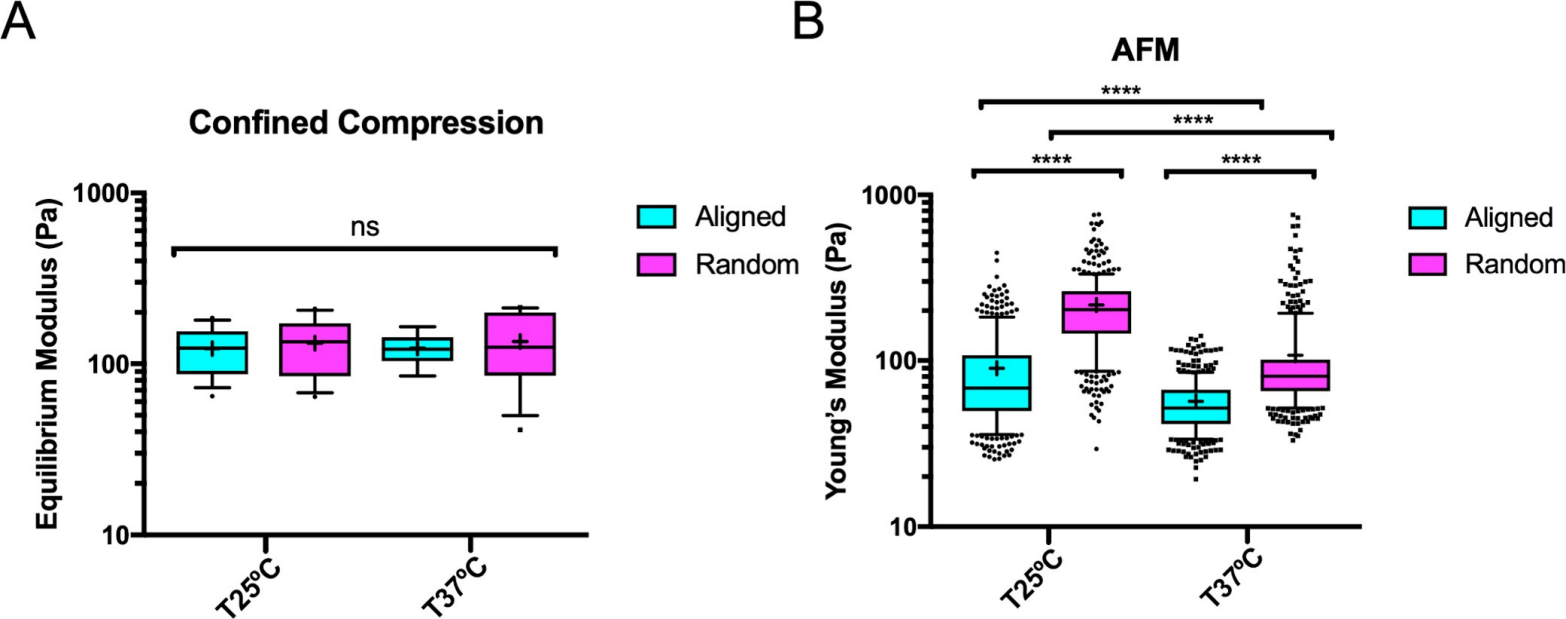
Mechanical properties of aligned and random collagen matrices at different temperatures. (A) Equilibrium modulus of gels measured by confined compression. Data presented as mean ± SEM. N = 8–16; n = 8–16. (B) Young’s modulus of gels measured by AFM. Data presented as median ± interquartile range (box), 10^th^-90^th^ percentile (whiskers), and mean (+) with outliers represented as points. N = 4; n = 335–379.

Collagen fiber architecture plays a significant role in determining the mechanical properties of collagen matrices [30]. Thus, to investigate the mechanical properties of the aligned collagen matrices, we utilized confined compression testing and atomic force microscopy to measure the micro- and macro-scale mechanical properties. Interestingly, confined compression measurements show that there were no significant differences in equilibrium modulus between aligned and randomly oriented matrices at 25°C or 37°C (Fig 3A). However, AFM measurements revealed a significant difference in stiffness between aligned and randomly oriented matrices at both temperatures, as well as significant differences in stiffness between matrices polymerized at 25°C or 37°C (Fig 3B). Notably, 25°C aligned and random matrices were significantly stiffer than their 37°C counterparts. Together, these findings reveal that macro-scale stiffness is not affected by collagen alignment; however, at the micro-scale, alignment affects stiffness independently of temperature.

## Discussion

Tumor progression brings about profound ECM remodeling, leading to distorted chemical and physical properties [2]. Importantly, physical properties of the tumor ECM, such as stiffness, have shown to be increasingly important during cancer progression [3]. As previously shown, physical properties of the ECM are highly dependent upon the architecture of the matrix [30,32–34]. A perturbed collagen architecture has been observed at the tumor periphery where cells have remodeled the ECM to form regions of highly aligned collagen fibers [15]. Furthermore, this architectural feature has been shown to have prognostic value in breast cancers [15] and provides guidance cues for cells escaping the primary tumor site [14]. As such, there have been significant efforts to investigate the role of collagen alignment during cancer progression and the underlying mechanisms by which aligned collagen accelerates cancer progression using 3D *in vitro* models [18,20,25,35]. However, the effects of collagen alignment on other features of the matrix that have known consequences, such as pore sizes and mechanical properties, have not been directly studied.

In this study, we used magnetic beads to align collagen matrices and assess the effects on pore size and macro- vs micro-scale mechanical properties. Quantification of collagen alignment revealed significant alignment at both 25°C and 37°C. However, there was disagreement between the quantification methods employed. The orientation index indicates no significant difference between alignment at 25°C and 37°C. In contrast, the aspect ratio indicates a higher degree of alignment at 25°C. We attribute this discrepancy to the underlying features each method uses to quantify the degree of alignment. In calculating the aspect ratio, the Fourier transform-based method evaluates the anisotropy of an entire image, while the orientation index is based on weighting the distribution of fiber angles. Our orientation index measurements indicate that a similar portion of aligned fibers at both temperatures are created, whereas aspect ratio measurements indicate that the aligned matrices are more anisotropic at 25°C compared to 37°C. This is likely due to lower polymerization temperatures inducing longer collagen fibers and thus enhancing the anisotropy of the images. These results illustrate a critical distinction between alignment quantification methods and emphasize the importance of understanding limitations of what can be concluded from the alignment analysis methods.

Architectural analysis revealed that collagen alignment resulted in temperature-dependent pore size differences. Specifically, we found that aligned collagen matrices at 37°C had significantly larger pore sizes than random matrices at 37°C. However, there was no significant difference in pore size between aligned and random gels at 25°C. Additionally, collagen matrices polymerized at 25°C were significantly stiffer than those polymerized at 37°C. Our results are in agreement with computational predictions by Stylianopolous et al. but disagree with experimental results from Ray et al [18]. However, the results reported by Ray et al. [18] are based on matrices aligned by cells, and it is possible that these matrices underwent additional remodeling aside from fiber alignment. Previous studies have shown that both alignment and pore size affect cancer cell migration [11,18]. Higher alignment has been shown to promote migration in the direction of alignment [18] and smaller pore sizes have been shown to hinder migration [11]. Thus, it is vital to fully understand the architectural properties of any experimental model being used to account for confounding architectural features, with our system displaying altered pore size with collagen alignment at 37°C.

Mechanical analysis revealed no significant differences in macro-scale stiffness but temperature independent differences in micro-scale stiffness. To measure macro- and micro-scale stiffness, we utilized confined compression and AFM, respectively. Confined compression revealed no difference in compressive moduli between aligned and random matrices at both temperatures (Fig 3A). This result is in agreement with Shannon et al. who used strong magnetic fields to align collagen matrices [35]. While they were unable achieve significant alignment at 37°C, they found no differences in compressive moduli between aligned and random gels across a range of lower temperatures [36]. It has been previously shown that macro-scale stiffness (as measured by unconfined compression) modulates epithelial cell behavior and induce a malignant phenotype [36]. However, macro-scale compression testing is not sufficient to detect mechanical differences in our system.

Micro-scale mechanical analysis via AFM revealed that aligned collagen matrices were significantly more compliant than their random counterparts at both temperatures (Fig 3B). Additionally, our AFM results also showed that matrices polymerized at 37°C were more compliant than their 25°C counterparts (Fig 3B). Strikingly, this contrasts our confined compression data (Fig 3A) that shows no differences between conditions. This is likely due to how compressive measurements at the macro- and micro-scale reflect different properties of the matrices. Macro-scale compressive testing is more dependent upon bulk architectural features such as density [29]. Micro-scale compressive testing via AFM measurements is more dependent upon features of individual collagen fibers or local fiber architecture. Prior studies have reported that polymerization temperature regulates fibril diameter, with lower temperatures creating larger diameter fibers and vice versa [24]. Thus, thicker fibers generated at lower temperatures may explain why our AFM measurements indicate both random and aligned collagen matrices polymerized at 25°C are significantly stiffer than their 37°C counterparts. Utilizing line scans from confocal reflectance images, we did not detect significant differences between fiber diameter amongst any of the conditions (S2 Fig). However, because this method is limited by the wavelength of light used to capture the confocal reflectance images, it is unable to accurately quantify features under 0.405 μm and prior reports indicate collagen fiber diameters under this constraint in the range of approximately 60–220 nm measured by scanning electron microscopy [24]. Nonetheless, our data is consistent with previous results indicating that larger diameter fibers are formed at lower temperatures [24] and larger fiber diameters lead to increased stiffness as measured by AFM [37].

While altered fiber diameter may explain the differences in stiffness between matrices polymerized at different temperatures, the change in stiffness observed between aligned and random matrices at a given temperature may be due to another local architecture parameter. Interconnectivity of the collagen network describes the extent of overlapping fibers in a cross-section and is a critical determinant of a network’s mechanical integrity [38]. Our data suggests alignment may reduce local network interconnectivity and thus explain our observed decreased stiffness in aligned matrices compared to their random counterparts at the same temperature [38]. While pore size was significantly larger in matrices polymerized at 25°C compared to their 37°C counterpart, there was only a significant difference between aligned and random matrices polymerized at 25°C. Thus, pore size does not appear to correlate with macro or micro-scale mechanics.

While it has become widely accepted that mechanical properties of the ECM drive cellular behavior that can contribute to cancer progression [39], it is less clear how architectural and mechanical properties at the micro- and macro-scale are related and how much each actually contribute to these phenomena. While it has become routine to measure the mechanical properties of 3D scaffolds, they do not report both micro- or macro-scale measurements [7,20–22]. Our experiments have revealed significant differences between micro- and macro-scale mechanical properties of aligned collagen matrices in addition to altered pore sizes. Collagen alignment is a prominent tumor associated collagen signature [13] and its full contribution to tumor progression is still unknown. Thus, as more aligned collagen scaffolds and tumor associated collagen signatures are investigated, it will be important to measure and consider the contribution of varying micro-scale mechanics and architecture and choose the scaffold conditions which hold the highest number of parameters constant. In our study, for example, it would be ideal to use collagen matrices polymerized at 25°C as they have similar pore sizes. These studies underscore the need to fully characterize all architectural and mechanical parameters of 3D culture systems to correctly identify the features responsible for driving cellular behavior without confounding variables.

## Supporting information

S1 Fig Collagen alignment system. (A) Fabrication of PDMS mold used to fabricate collagen matrices. (B) Schematic depicting the alignment of collagen fibers via magnetic beads. (C) An image of an aligned collagen gel in a fabricated PDMS mold.
(TIFF)

S2 Fig Collagen fiber diameters. Fiber diameter of matrices measured using line scans from confocal reflectance images. Data presented as median ± interquartile range (box), 10^th^-90^th^ percentile (whiskers), and mean (+) with outliers represented as points. N = 6–7; n = 36–42.
(TIFF)
