# Supplementary figures and images for: Fiber alignment drives changes in architectural and mechanical features in collagen matrices
Source: PLoS One. 2019 May 15;14(5):e0216537. doi: 10.1371/journal.pone.0216537 (PMC6519824; doi:10.1371/journal.pone.0216537)

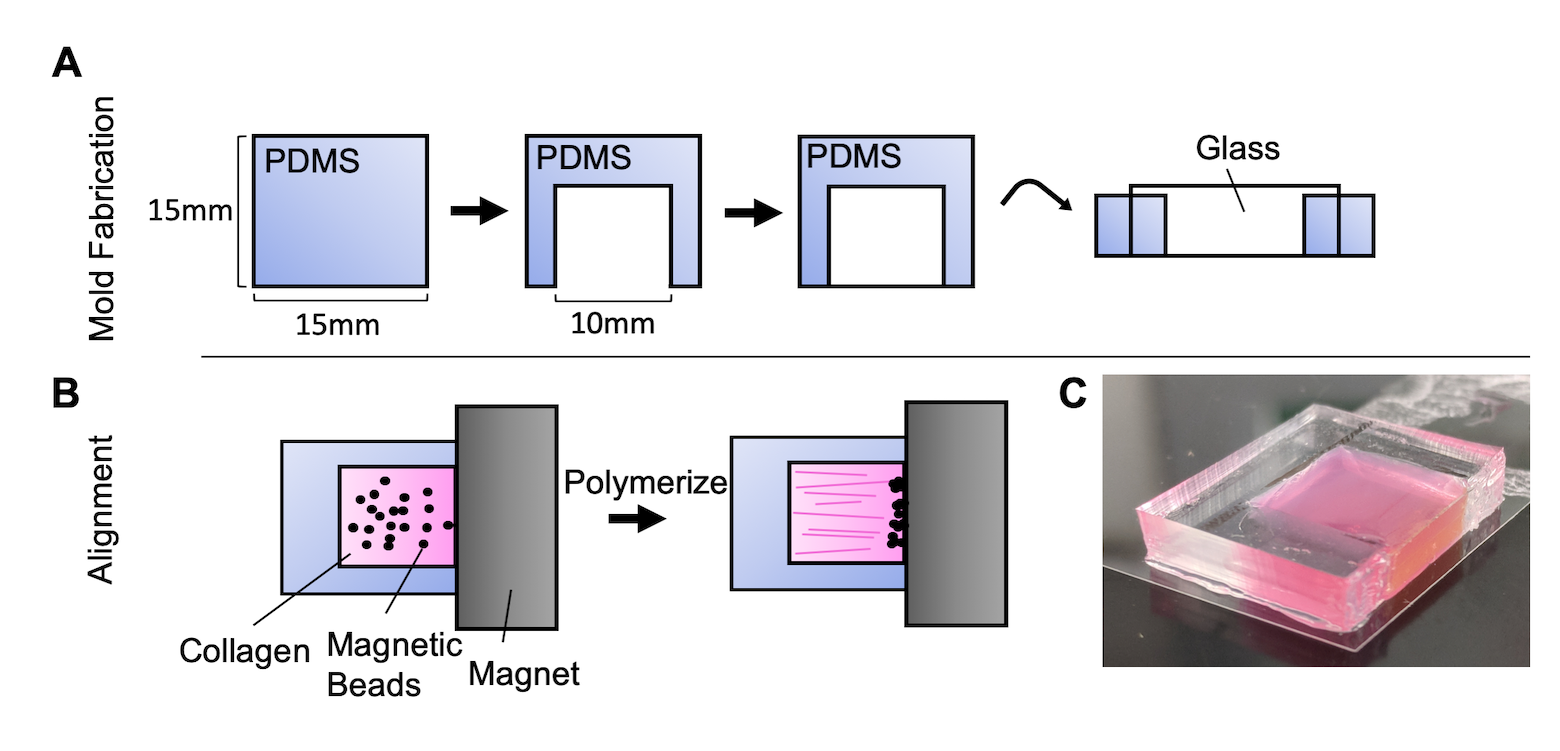

Supplement: S1 Fig — (A) Fabrication of PDMS mold used to fabricate collagen matrices. (B) Schematic depicting the alignment of collagen fibers via magnetic beads. (C) An image of an aligned collagen gel in a fabricated PDMS mold. (TIFF) [file pone.0216537.s001.tiff]

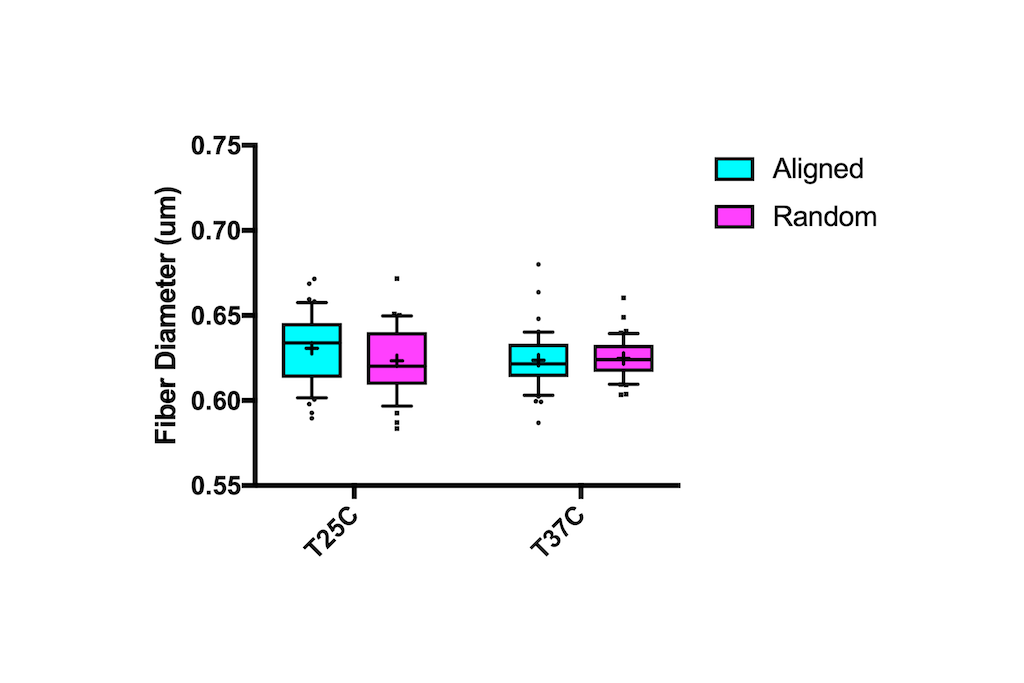

Supplement: S2 Fig — Fiber diameter of matrices measured using line scans from confocal reflectance images. Data presented as median ± interquartile range (box), 10th-90th percentile (whiskers), and mean (+) with outliers represented as points. N = 6–7; n = 36–42. (TIFF) [file pone.0216537.s002.tiff]
